# Supplementary material for: Reconciling Mining with the Conservation of Cave Biodiversity: A Quantitative Baseline to Help Establish Conservation Priorities
Source: PLoS One. 2016 Dec 20;11(12):e0168348. doi: 10.1371/journal.pone.0168348 (PMC5173368; doi:10.1371/journal.pone.0168348)
Supplement: S1 Dataset — (ZIP) [file pone.0168348.s002.zip › Taxa/Serra Norte/SN_2013/N4WS/Lista N4WS-25.pdf]

CAVIDADE N4WS-0025

Classe	Ordem	Fam/Outros	Gên/Outros	Espécie	S-U	U-S	Zon.
Arachnida	Amblypygi	Phrynidae	*Heterophrynus*	*longicornis*	1		E
Arachnida	Araneae	Ctenidae	*Ctenus*	sp.2		1	P
Arachnida	Araneae	Pholcidae	*Mesabolivar*	*aurantiacus*	X	X	E/P
Arachnida	Araneae	Scytodidae	*Scytodes*	jovem	8	10	E/P
Arachnida	Araneae	Tetragnathidae	*Leucauge*	sp.1	X		E
Arachnida	Opiliones	Sclerosomatidae	*Prionostema*	sp.1	X	X	E/P
Arachnida	Opiliones	Stygnidae	*Protimesius*	*gracilis*	1		E
Entognatha	Collembola	Paronellidae		sp.1	X	X	E
Insecta	Blattodea	Polyphagidae		jovem	4		E
Insecta	Coleoptera	Staphylinidae		sp.66		X	P
Insecta	Coleoptera	Tenebrionidae		jovem	X		E
Insecta	Diptera	Calliphoridae	Mesembrinellinae	sp.		X	P
Insecta	Diptera	Cecidomyiidae	Cecidomyiinae	sp.	X		E
Insecta	Diptera	Ceratopogonidae		jovem	X		E
Insecta	Diptera	Chironomidae		sp.	X	X	E
Insecta	Diptera	Culicidae	*Culex*	sp.		X	P
Insecta	Diptera	Diastatidae		sp.	X		E
Insecta	Diptera	Drosophilidae		sp.	X		E
Insecta	Diptera	Psychodidae	*Sciopemyia*	*sordellii*		X	P
Insecta	Diptera	Tipulidae	Tipulinae	sp.	X		E
Insecta	Heteroptera	Reduviidae	Reduviinae	jovem	1		E
Insecta	Hymenoptera	Formicidae	*Camponotus*	*atriceps*		1	E
Insecta	Hymenoptera	Formicidae	*Crematogaster*	sp.1	X		E
Insecta	Hymenoptera	Formicidae	*Dolichoderus*	sp.1	X		E
Insecta	Hymenoptera	Formicidae	*Nylanderia*	sp.1	X		E
Insecta	Hymenoptera	Formicidae	*Nylanderia*	sp.2	X		E
Insecta	Hymenoptera	Formicidae	*Pachycondyla*	*striata*		3	E/P
Insecta	Hymenoptera	Formicidae	*Pheidole*	sp.2	X		E
Insecta	Lepidoptera	Noctuoidea		sp.1	1	4	P
Insecta	Lepidoptera	Noctuoidea		sp.15	1		E
Insecta	Orthoptera	Phalangopsidae	*Paraclodes*	sp.	4	1	E
Insecta	Orthoptera	Phalangopsidae	*Phalangopsis*	sp.	5	7	E/P
Insecta	Orthoptera	Phalangopsidae		sp.1		2	E/P
Insecta	Psocoptera	Psocomorpha		jovem	X		E
Insecta	Thysanoptera	Thripidae		sp.1	X		E
Malacostraca	Isopoda	Armadillidae		sp.1	X		E
Amphibia	Anura	Strabomantidae	*Pristimantis*	cf. *fenestratus*	3		E
Mammalia	Chiroptera	Emballonuridae	*Peropteryx*	sp.	1	5	E
Reptilia	Squamata			sp.2	1		E
